# The Anti-Inflammatory Effects and Mechanisms of Eupafolin in Lipopolysaccharide-Induced Inflammatory Responses in RAW264.7 Macrophages

**DOI:** 10.1371/journal.pone.0158662

**Published:** 2016-07-14

**Authors:** Chin-Chaun Chen, Ming-Wei Lin, Chan-Jung Liang, Shu-Huei Wang

**Affiliations:** 1 Graduate Institute of Natural Products, Chang Gung University, Taoyuan, Taiwan; 2 Chinese Herbal Medicine Research Team, Healthy Aging Research Center, Chang Gung University, Taoyuan, Taiwan; 3 Tissue Bank, Chang Gung Memorial Hospital, Taoyuan, Taiwan; 4 Center for Lipid and Glycomedicine Research (CLGR), Kaohsiung Medical University, Kaohsiung, Taiwan; 5 Center for Lipid Biosciences (CLB), Kaohsiung Medical University Hospital, Kaohsiung, Taiwan; 6 Department of Anatomy and Cell Biology, College of Medicine, National Taiwan University, Taipei, Taiwan; National Institutes of Health, UNITED STATES

## Abstract

Eupafolin is a flavone isolated from *Artemisia princeps* Pampanini (family Asteraceae). The aim of this study was to examine the anti-inflammatory effects of eupafolin in lipopolysaccharide (LPS)-treated RAW264.7 macrophages and LPS-induced mouse skin and lung inflammation models and to identify the mechanism underlying these effects. Eupafolin decreased the LPS-induced release of inflammatory mediators (iNOS, COX-2 and NO) and proinflammatory cytokines (IL-6 and TNF-α) from the RAW264.7 macrophages. Eupafolin inhibited the LPS-induced phosphorylation of p38 MAPK, ERK1/2, JNK, AKT and p65 and the nuclear translocation of p65 and c-fos. These effects were mainly mediated by the inhibition of JNK. In the mouse paw and lung models, eupafolin effectively suppressed the LPS-induced edema formation and down-regulated iNOS and COX-2 expression. These results demonstrated that eupafolin exhibits anti-inflammatory properties and suggested that eupafolin can be developed as an anti-inflammatory agent.

## Introduction

Inflammation is a physiological response against harmful stimuli, such as pathogens, in the body. By inducing the release of signaling molecules, inflammation exerts protective effects that neutralize injurious pathogens. Macrophages are important cells of the immune system that act as the first line of defense against invading agents (bacteria, viruses, and fungi) and respond to pathogen attacks by releasing cellular signaling molecules and various proinflammatory cytokines, including tumor necrosis factor-α (TNF-α), interferon-γ (IFN-γ), interleukin 1-β (IL-1β) and IL-6, and inflammatory mediators, such as nitric oxide (NO), prostaglandin E_2_ (PGE_2_), and cyclooxygenase-2 (COX-2)[1–3]. However, chronic inflammation and deregulated cytokine production are associated with conditions such as cancer progression, cardiovascular disease, diabetes, and obesity[4–6]. Therefore, one effective strategy for developing therapeutic agents to treat severe inflammation is the identification of agents that regulate the production of proinflammatory mediators.

Eupafolin (6-methoxy-5,7,3′,4′-tetrahydroxyflavone) is the major active flavonoid isolated from *Artemisia princeps* Pampanini (family Asteraceae)[7, 8]. Previous reports have shown that eupafolin has several biological effects. For example, eupafolin exerts anti-inflammatory effects on TNF-treated A549 (lung cancer) cells and on lipopolysaccharide (LPS)-stimulated RAW264.7 cells [9, 10] and anti-proliferative effects on B16-F10 (murine melanoma)[11] and HeLa (human cervical adenocarcinoma) cells[12]. However, no reports elucidating the molecular mechanism underlying its anti-inflammatory effects have been published.

The aim of present study was to evaluate the potential anti-inflammatory activities of eupafolin by quantifying its inhibitory effects on iNOS and COX2 expression in LPS-activated RAW264.7 macrophages and in the LPS-induced mouse paw edema and acute lung injury (ALI) models. Our results showed that eupafolin has strong anti-inflammatory effects *in vitro* and *in vivo* and revealed the involvement of NF-κB, AP-1 and JNK.

## Materials and Methods

### Reagents

Polyclonal rabbit IgGs against human GAPDH, β-actin, phospho/total-p38, phospho/total-ERK1/2, phospho/total-JNK, COX-2, and phospho/total -AKT and horseradish peroxidase (HRP)-conjugated goat anti-mouse IgG or anti-rabbit IgG antibodies were purchased from GeneTex (Irvine, CA, USA). Monoclonal rabbit antibodies against human total-p65 and phospho-p65 were purchased from GeneTex. Monoclonal rabbit antibodies against human Proliferating Cell Nuclear Antigen (PCNA) were purchased from Santa Cruz Biotechnology (Santa Cruz, CA). Polyclonal mouse IgG against human c-fos and iNOS were purchased from Cell Signaling (Beverly, MA, USA). LY294002, PD98058, SP600125, and SB203580 were purchased from Biomol (Plymouth Meeting, PA, USA). Polyvinylidene difluoride (PVDF) membranes were purchased from Millipore (Billerica, MA, USA). LPS and BrdU were purchased from Sigma–Aldrich (St. Louis, MO, USA). TRITC and FITC-conjugated goat anti-mouse IgGs were purchased from Jackson ImmunoResearch (West Grove, Pennsylvania, USA).

### Extraction and purification of eupafolin

**Fig 1:**
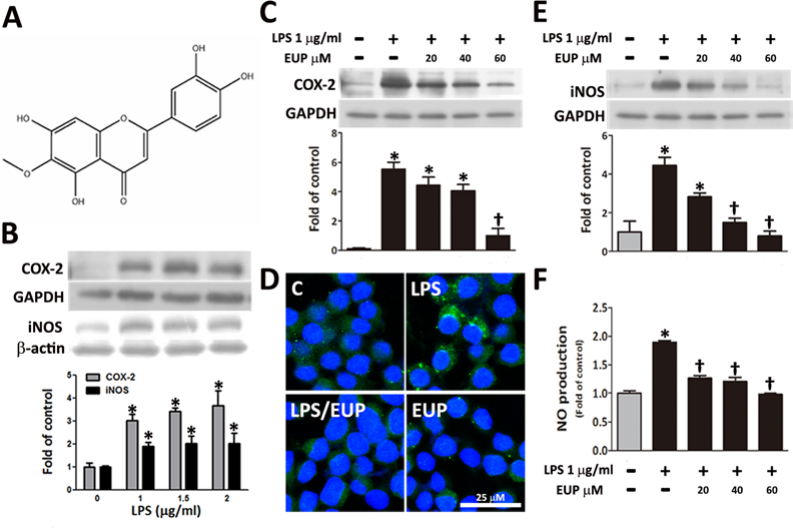
Eupafolin inhibited the LPS-induced NO, iNOS and COX-2 expression in RAW264.7 macrophages. (A) Chemical structure of eupafolin. RAW264.7 macrophages were pretreated with 1, 1.5, or 2 μg/ml of LPS for 24 h. The control group included cells grown in medium without LPS. (B) COX-2 and iNOS protein expression was determined by Western blot analysis. GAPDH or β-actin was processed in parallel as an internal control for protein loading. (C) The RAW264.7 macrophages were pretreated with 0, 20, 40, or 60 μM eupafolin for 1 h and then with 1 μg/ml of LPS for another 24 h. The COX-2 expression was analyzed by Western blotting (n = 4). (D) Immunofluorescence staining was performed to show the COX-2 expression. (n = 4) (E) The RAW264.7 macrophages were pretreated with 0, 20, 40, or 60 μM eupafolin for 1 h and then with 1 μg/ml of LPS for another 24 h. The iNOS expression was measured using Western blot analysis (n = 4). (F) The Griess assay was performed to evaluate the NO production. In B-C and E-F, the data are shown as the means ± SEM (n = 4). *P<0.05 vs. the untreated group; ^†^*P*<0.05 vs. the LPS-treated group. The scale bars in D = 25 μm.

Eupafolin was kindly provided by Dr. H. H. Ko and F. L. Yen. Briefly, the dried aerial parts of *P*. *nodiflora* (7.5 kg) were dissolved in 1 l of methanol at room temperature. This extraction procedure was repeated 3 times. The methanolic extract was collected, filtered, concentrated, and then suspended in water and successively portioned with equivalent volumes of n-hexane, chloroform, and ethyl acetate. The ethyl acetate-soluble fraction was subjected to silica gel column chromatography and purified with a mixture of n-hexane and ethylacetate in a 1:8 ratio, followed by purification with methanol. The purified fraction was recrystallized to obtain 756 mg of eupafolin (Fig 1A), and the purified eupafolin was stored at -20°C until further use. Eupafolin (> 97% purity) were extracted and analyzed by HPLC.

### Cell culture

RAW264.7 macrophages, obtained from the American Type Culture Collection (MD, USA), were cultured in DMEM containing 10% FBS and 1% antibiotic/antimycotic solution at 37°C in a 5% CO_2_ atmosphere.

### Cell viability

RAW264.7 macrophages were treated with various concentrations of eupafolin or LPS for 24 h, and the cell viability was determined using the 3-(4,5-dimethylthiazol-2-yl)-2,5-diphenyl tetrazolium bromide (MTT) assay.

### Nitrite determination and prostaglandin E2 (PGE2) assay

Nitric oxide secretion was determined using the Griess assay. Briefly, the RAW264.7 macrophages were treated with various concentrations of eupafolin for 1 h or with PD98059 (an ERK inhibitor), SB203580 (a p38 inhibitor) or SP600125 (a JNK inhibitor) for 30 min before treatment with LPS (1 μg/ml) for 24 h. The culture media were collected and mixed with an equal amount of Griess reagent in a 96-well plate. The absorbance at 550 nm was determined using an ELISA plate reader.

The prostaglandin E2 concentration in the cell culture medium was determined using a PGE_2_ assay kit (Cayman Chemical, Ann Arbor, MI, USA). All procedures were performed according to the manufacturer’s instructions.

### Mouse cytokine array

RAW264.7 macrophages were pretreated with eupafolin (60 μM) for 1 h and then stimulated with LPS (1 μg/ml) for 24 h. The conditioned medium and cell lysates were collected for cytokine secretion analysis. The cytokine expression profiles were determined using mouse cytokine membrane array (R&D System Inc., Minneapolis, MN, USA) according to the manufacturer's instructions.

### Immunofluorescence staining

RAW264.7 macrophages, which were plated on cover glasses, were pretreated with eupafolin for 1 h and then treated with LPS for the indicated times. After treatment, the cells were fixed with 4% paraformaldehyde for 30 min and then permeabilized with 0.05% Triton X-100 for 2 min. The fixed cells were blocked in 10% normal goat serum for 1 h and then incubated with the primary antibodies (all at 1:100 in blocking solution) at 4°C overnight. Then, the cells were incubated with the FITC-conjugated secondary antibody for 1 h at room temperature. The cells were counterstained with DAPI and examined using a fluorescence microscope.

### Immunoblotting analysis

The RAW264.7 macrophages were stimulated with LPS alone or together with eupafolin. The cells were lysed with lysis buffer (RIPA, Cell Signaling, Beverly, MA, USA). The proteins (20 μg) were subjected to SDS–PAGE on 10–12% polyacrylamide gradient gels and transferred to PVDF membranes (Millipore, Bedford, MA). The membranes were blocked with 5% BSA at room temperature for 1 h. The blots were then incubated with the primary antibodies (all at 1:1000 in 1.5% BSA) at 4°C overnight and then incubated with the HRP-conjugated secondary antibodies (all at 1:3000) at room temperature for 1 h. The immunoreactivity was detected with ECL (GE Healthcare Bioscience). The intensities of the bands were quantified using Gel-Pro software. Rabbit anti-human GAPDH and β-actin antibodies were used as internal controls (all at 1:3000 in 1.5% BSA). The protein quantities were normalized to the intensities of the internal control bands.

### Nuclei extraction

RAW264.7 macrophages were pretreated with eupafolin for 1 h and then treated with LPS for the 30 min. The nuclear fractions were isolated using the NE-PER Kit (Pierce Biotech, Rockford, IL) according to the user guide provided by the manufacturer.

### Knockdown of gene expression

Knockdown of AKT, JNK and P38 gene expression was performed by transfection with small interfering RNAs (siRNAs). The RAW264.7 macrophages (5x10^6^ cells) were added to 100 μl of Nucleofector solution (Lonza, Allendale, NJ), and the JNK-, p38-siRNA oligomers (1 μM) (Invitrogen, CA, USA) or AKT- siRNA oligomers (1 μM) (Santa Cruz, CA) were electroporated according to the manufacturer’s instructions. The cells were transfected for 48 h. The siRNA effects were evaluated by Western blotting.

### LPS-induced paw edema test in mice

Male C57BL/6J (8 w) mice were purchased from the National Laboratory Animal Center (Taipei, Taiwan). All procedures were performed in accordance with the local institutional guidelines for animal care of the National Taiwan University and complied with the “Guide for the Care and Use of Laboratory Animals” NIH publication No. 86–23, revised 2011. The protocol was approved by the National Taiwan University College of Medicine and College of Public Health Institutional Animal Care and Use Committee (IACUC NO: 20150293). The mice were divided into 3 groups, which were given the following: (1) 50 μL of PBS as a control, (2) 1 mg/kg body weight of LPS in 50 μL of PBS, and (3) 10 mg/kg eupafolin plus 1 mg/kg body weight of LPS in 50 μL of PBS. The 10 mg/kg eupafolin was administered i.p. 30 min before the LPS administration. After 24 h treatment with LPS, the mice were sacrificed by an overdose of pentobarbital and the paws were collected and fixed in 4% paraformaldehyde, paraffin-embedded, and cross-sectioned for morphometric analysis and immunohistochemistry. The paws were cut serially into 5 μm sections, and every tenth section was stained with hematoxylin and eosin (Sigma). For morphometric analysis of the edema of the paws, the mean edema was calculated by determining the distance between the epidermis and the muscle layer.

### LPS-induced ALI mouse model

The mice were randomly divided into three groups: (1) 50 μl of PBS as a control, (2) 0.2 mg/kg body weight of LPS in 50 μl of PBS, and (3) 25 mg/kg eupafolin plus 0.2 mg/kg body weight of LPS in 50 μl of PBS. The 25 mg/kg eupafolin was pre-administered i.p. for 2 days and then 25 μl of LPS (0.2 mg/kg) was instilled intranasally in each nostril to induce lung injury. The mice were sacrificed 24 h after the LPS treatment by an overdose of pentobarbital, and then ice-cold PBS (5 ml) was administered to the lung to obtain the bronchoalveolar lavage fluid (BALF). The BALF was centrifuged, and each cell pellet was stained with Trypan blue (Sigma) and counted in a hemacytometer to determine the total cell number in the BALF. The lung tissue was fixed in 4% paraformaldehyde, paraffin-embedded, and cross-sectioned for morphometric analysis and immunohistochemistry.

### Statistical analyses

All values are provided as the means ± SEM. Statistical comparisons were performed using Student’s t-test and one-way ANOVA. Significance was defined as *P-*values*<*0.05.

## Results

### Effect of eupafolin on LPS-induced NO, iNOS and COX-2 expression in RAW264.7 macrophages

LPS is well known to induce the secretion of inflammatory cytokines[13–15]. To determine the appropriate dose of LPS for use in the chemokine secretion study, we investigated the effect of LPS (0, 0.5, 1, 1.5, 2, and 2.5 μg/ml) on the proliferation of the RAW264.7 macrophages using the MTT assay. The cell viability was, respectively, 1.04±0.03, 1.05±0.02, 1.02±0.03, 0.99±0.03, or 0.94±0.02 of control levels. The results showed that LPS exhibits no proliferative or cytotoxic effects on the RAW264.7 cells. To exclude the possibility that the anti-inflammatory effect of eupafolin was due to cytotoxicity to the RAW264.7 macrophages, the MTT test was performed. A range of concentrations of eupafolin (0, 20, 40, 60, 80, and 100 μM) was used in this study. The cell viability was, respectively, 1.04±0.02, 1.03±0.02, 0.97±0.02, 0.97±0.03, or 0.87±0.03 of control levels. The results showed that 0–80 μM eupafolin was not cytotoxic to the RAW264.7 macrophages. However, at a concentration of 100 μM, eupafolin was cytotoxic to the RAW264.7 macrophages (from 1.00±0.02 to 0.87±0.03). To determine whether eupafolin had an anti-inflammatory effect, we examined the expression of COX-2 and iNOS in the LPS-induced RAW264.7 macrophages. The results showed that treatment with the various concentrations of LPS induced the expression of COX-2 and iNOS (Fig 1B). Furthermore, the increases were significantly reduced in a dose-dependent manner by the eupafolin pretreatment (Fig 1C–1F). Therefore, doses of 1 μg/ml of LPS and 60 μM eupafolin were used in the subsequent experiments.

### Effect of eupafolin on LPS-induced cytokine expression in RAW264.7 macrophages

**Fig 2:**
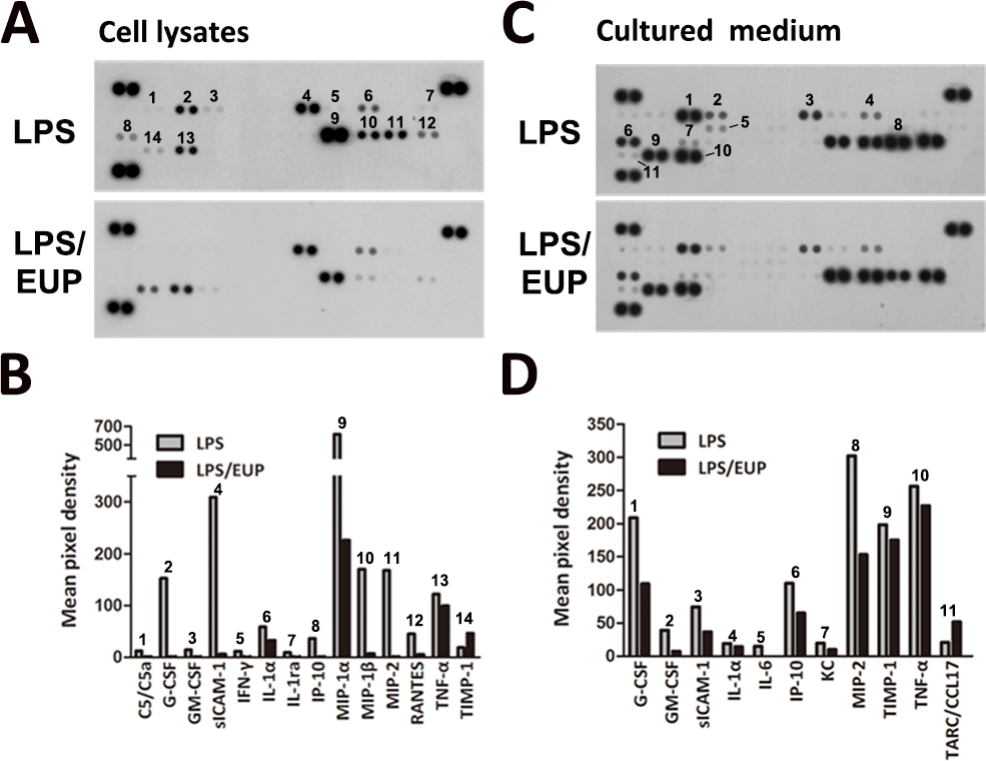
Eupafolin inhibited the LPS-induced cytokine expression in RAW264.7 macrophages. The RAW264.7 macrophages were pretreated with 60 μM eupafolin for 1 h and then with 1 μg/ml of LPS for another 24 h. The cytokine expression was regulated by LPS and eupafolin treatment. Protein extracts (A) and cultured medium (C) were collected to perform the cytokine membrane array assay. (B, D) Quantification of the cytokine expression.

To assess the anti-inflammatory properties of eupafolin in RAW264.7 macrophages, we first assessed the effect of eupafolin treatment on cytokine production in LPS-stimulated RAW264.7 macrophages. The cell lysates and culture medium were collected for cytokine array detection. The results of this assay indicated that the treatment with 60 μM eupafolin significantly decreased the levels of multiple cytokines in LPS-treated RAW264.7 macrophages (Fig 2). Specifically, the high expression levels of G-CSF, GM-CSF, IL-6, sICAM-1, MIP-1, MIP-2, IP-10 and TNF-ɑ in LPS-treated RAW264.7 macrophages were dramatically reduced by the eupafolin treatment. These results indicate that eupafolin can inhibit LPS-induced inflammatory responses in RAW264.7 macrophages.

### Effects of eupafolin on LPS-induced activation of MAPKs in RAW264.7 macrophages

**Fig 3:**
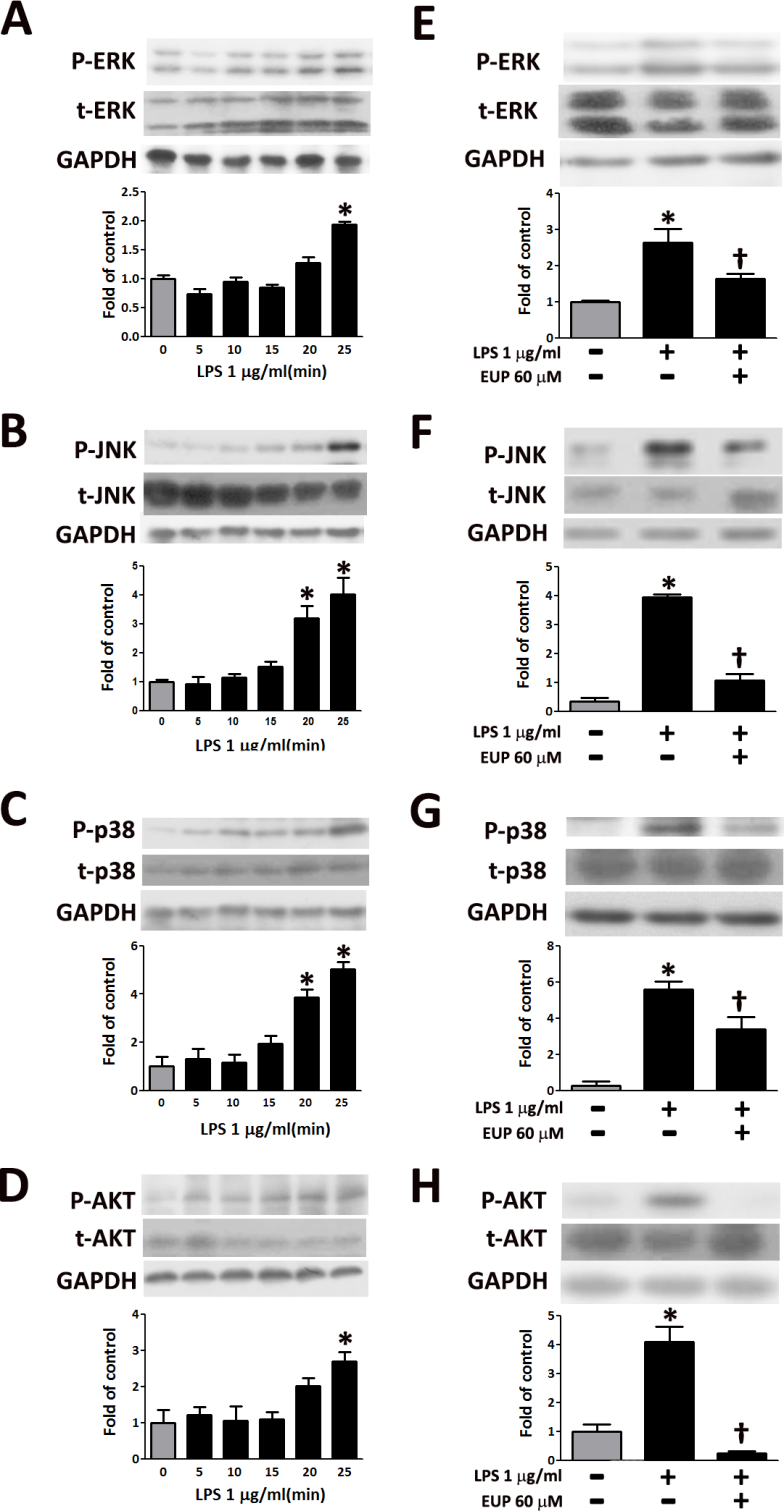
Eupafolin inhibited the LPS-induced activation of MAPKs in RAW264.7 macrophages. The RAW264.7 macrophages were pretreated with 1 μg/ml of LPS for various times as indicated. The phosphorylated and total (A) ERK, (B) JNK, (C) p38, or (D) AKT levels were determined by Western blot analysis. Total ERK (t-ERK), total JNK (t-JNK), total p38 (t-p38), or total AKT (t-AKT) protein was used as the loading control. The data are shown as the means ± SEM (n = 6). *P<0.05 vs. the untreated control. The cells were treated for 1 h with 60 μM eupafolin and were then incubated with 1 μg/ml of LPS for 30 min. The phosphorylated (E) ERK, (F) JNK, (G) p38, or (H) AKT levels were determined by Western blot analysis. Total ERK (t-ERK), total JNK (t-JNK), total p38 (t-p38), or total AKT (t-AKT) protein was used as the loading control. The data are shown as the means ± SEM (n = 6). *P<0.05 vs. the untreated control, ^†^*P*<0.05 vs. the LPS-treated cells.

Previous studies have shown that MAPKs and AKT are involved in the signal transduction pathways that lead to the regulation of inflammatory mediators[16–18]. Therefore, we investigated the effects of eupafolin on the LPS-induced phosphorylation of MAPKs and AKT in RAW264.7 macrophages by Western blotting. As shown in Fig 3A–3D, the phosphorylation levels of MAPKs and AKT in RAW264.7 macrophages were significantly increased by the LPS treatment in a time-dependent manner. Compared with the expression in the LPS-induced group, the LPS-induced phosphorylation of p38, JNK, ERK and AKT was suppressed by eupafolin pre-treatment (Fig 3E–3H). These results indicate that phosphorylation of p38, JNK, ERK and AKT may be involved in the inhibition of the LPS-induced inflammatory responses in RAW264.7 macrophages.

### Effects of eupafolin on LPS-induced NO, iNOS, COX-2 and PGE_2_ expression in RAW264.7 macrophages are mediated by MAPK activation

Specific MAPK inhibitors were used to further confirm that the anti-inflammatory effect of eupafolin on the LPS-induced NO, iNOS, COX-2 and PGE_2_ expression in RAW264.7 cells involves the MAPK signal transduction pathways.

**Fig 4:**
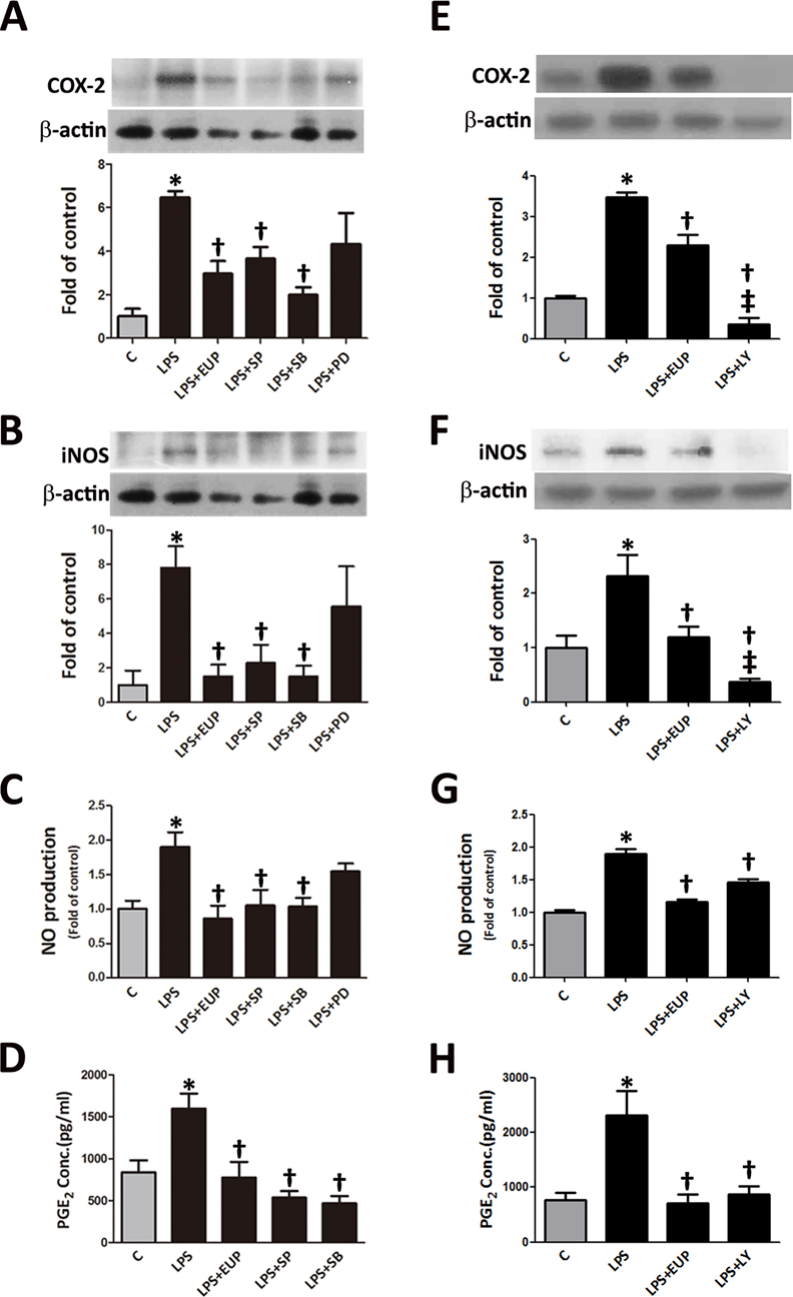
The eupafolin-mediated inhibition of the LPS-induced NO, iNOS, COX-2 and PGE_2_ expression in RAW264.7 macrophages involved MAPK activation. The RAW264.7 macrophages were treated for 1 h with 30 μM of the MAPK inhibitors or 10 μM of the PI3K/AKT inhibitor and were then incubated with 1 μg/ml of LPS for 24 h. (A) COX-2 and (B) iNOS protein expression was determined by Western blot analysis. β-actin was processed in parallel as an internal control for protein loading. (C) NO was measured with the Griess assay, and (D) PGE_2_ was measured with an ELISA assay. The data are shown as the means ± SEM (n = 5–8). *P<0.05 vs. the untreated control, ^†^*P*<0.05 vs. the LPS-treated cells. ^‡^*P*<0.05 vs. the LPS+EUP treated cells.

**Fig 5:**
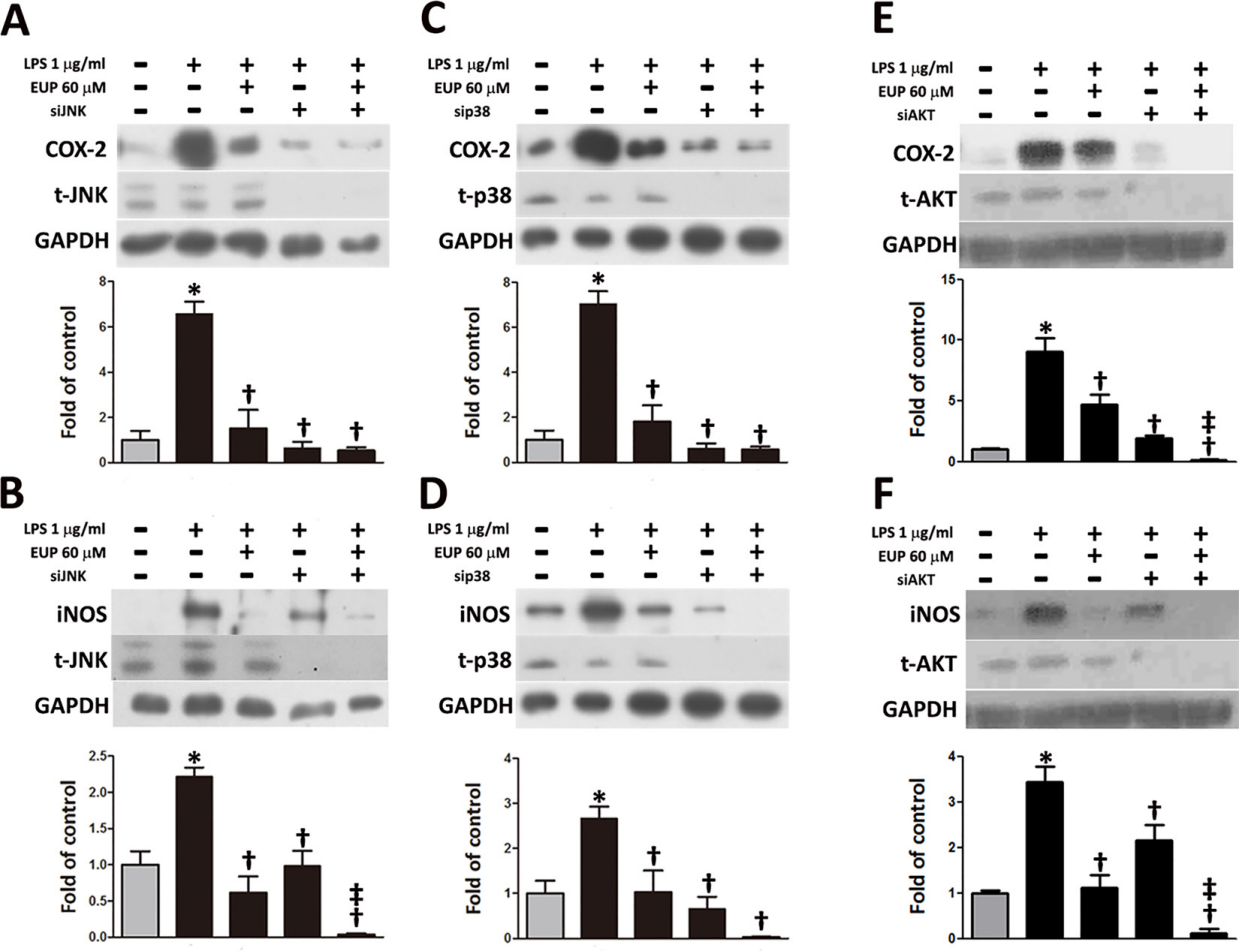
Effects of p38-, JNK- and AKT-specific siRNA on the COX-2 and iNOS expression in LPS-treated RAW264.7 macrophages. The expression of (E, G) COX-2 and (F, H) iNOS was determined by Western blot analysis after p38 or JNK silencing followed by LPS treatment. p38-, JNK- and AKT-specific siRNA reduced the total p38 (t-p38), JNK (t-JNK) and AKT (t-AKT) protein expression, respectively, compared with that in the non-treated cells. The values are shown as the means ± SEM (n = 3–6). *P<0.05 vs. the untreated control, ^†^*P*<0.05 vs. the LPS-treated cells. ^‡^*P*<0.05 vs. the LPS+EUP treated cells.

As shown in Fig 4A–4H, LY294002 (a PI3K/AKT inhibitor) and specific MAPK inhibitors, including SP600125 (a JNK inhibitor) and SB302580 (a p38 inhibitor), significantly suppressed the LPS-stimulated increases in the levels of COX-2, iNOS, NO, and PGE_2_, but PD98059 (an ERK inhibitor) was ineffective. Similar effects were observed in the siRNA experiments: when the expression of JNK, p38 or AKT was blocked, the LPS-stimulated expression of COX-2 and iNOS was significantly reduced (Fig 5A–5F). These results indicate that eupafolin suppressed the LPS-induced changes in the expression of inflammatory molecules in RAW264.7 macrophages by inhibiting the activation of JNK, p38 or AKT.

### Effects of eupafolin on LPS-induced NF-κB p65 and AP-1/c-fos nuclear translocation in RAW264.7 macrophages

**Fig 6:**
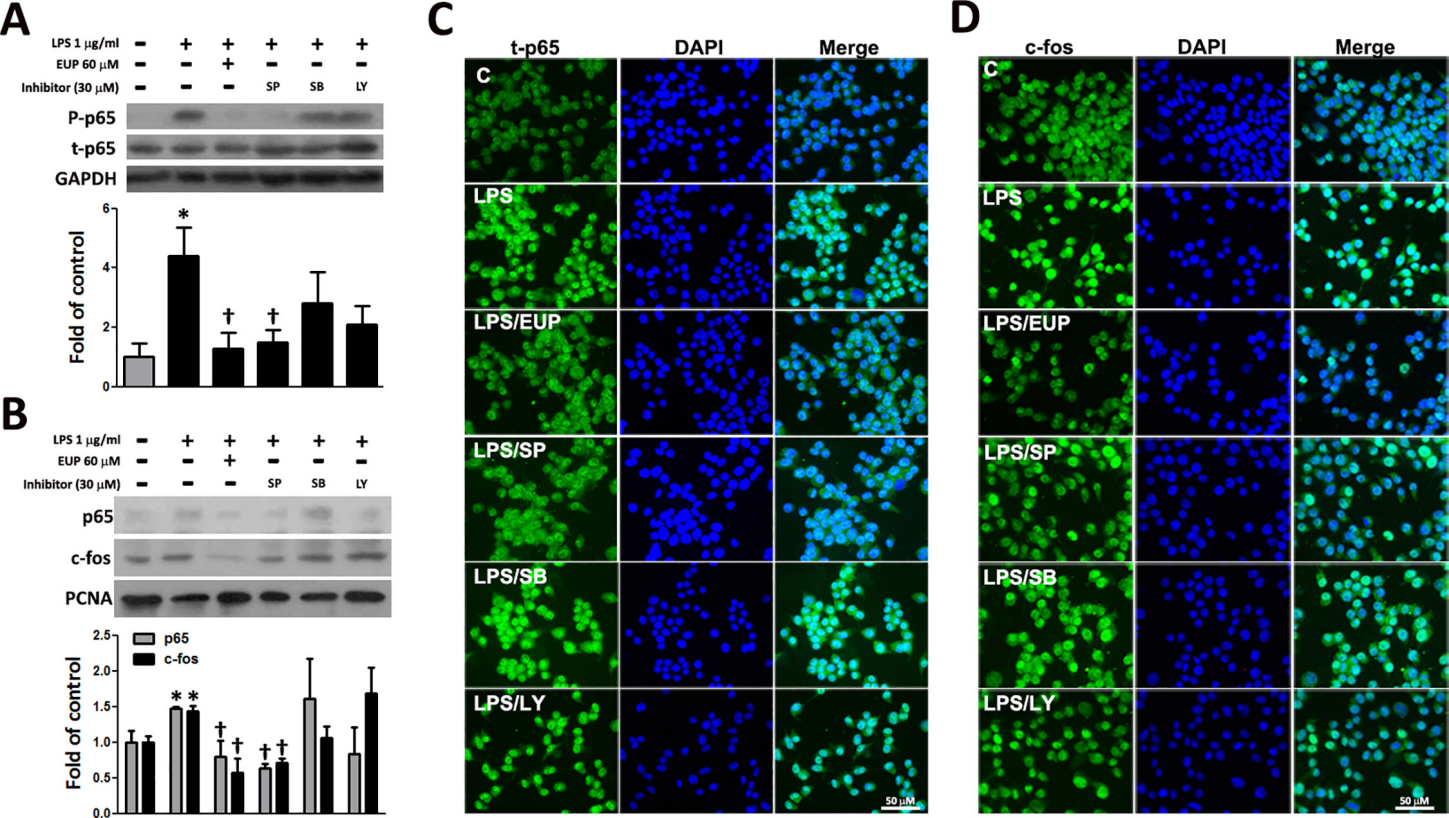
Effects of eupafolin on the LPS-induced NF-κB p65 and AP-1/c-fos nuclear translocation in RAW264.7 macrophages. The RAW264.7 macrophages were pretreated for 1 h with 60 μM eupafolin or with 30 μM of the MAPK inhibitors and were then treated with 1 μg/ml of LPS for 30 min. Western blot analysis (n = 3) was performed to evaluate the subcellular and nuclear localization of (A) phosphorylated (P-p65) and total p65 (t-p65) (total cell lysate) and (B) p65 and c-fos (nuclear fraction). GAPDH and PCNA (proliferating cell nuclear antigen) were processed in parallel as internal controls for protein loading. *P<0.05 vs. the untreated control, ^†^*P*<0.05 vs. the LPS-treated cells. Immunofluorescence staining (n = 3) was performed to show the subcellular localization of (C) t-p65 and (D) c-fos. Bar = 50 μm.

The MAPK signaling pathway plays an important role in regulating the activity of nuclear factor-*kappa B* (NF-κB) and activator protein 1 (AP-1)[19]. NF-κB and AP-1 are important transcription factors that regulate the expression of most proinflammatory cytokines as well as the levels of iNOS, COX-2, and PGE_2_[20–22]. To evaluate whether the anti-inflammatory effect of eupafolin acted through the NF-κB and AP-1 pathways, the phosphorylation of the p65 subunit of NF-κB and the nuclear translocation of p65 and the c-fos subunit of AP-1 were examined by Western blot and immunofluorescence staining. As shown in Fig 6A–6D, the amounts of p65 and c-fos in the nuclei markedly increased following exposure to LPS alone; however, the LPS-induced increases in the p65 and c-fos levels in the nuclear fraction were reduced following eupafolin pretreatment. Furthermore, these suppressive effects of eupafolin in the LPS-treated RAW264.7 cells were the same in the LPS-treated RAW264.7 cells by SP600125 pretreatment but not by SB203580 and LY294002. Based on these results, we found that eupafolin may significantly inhibit NF-κB and AP-1 activation in LPS-stimulated RAW264.7 cells through a JNK-dependent pathway.

### Effects of eupafolin on LPS-induced paw edema and inflammation in mice

**Fig 7:**
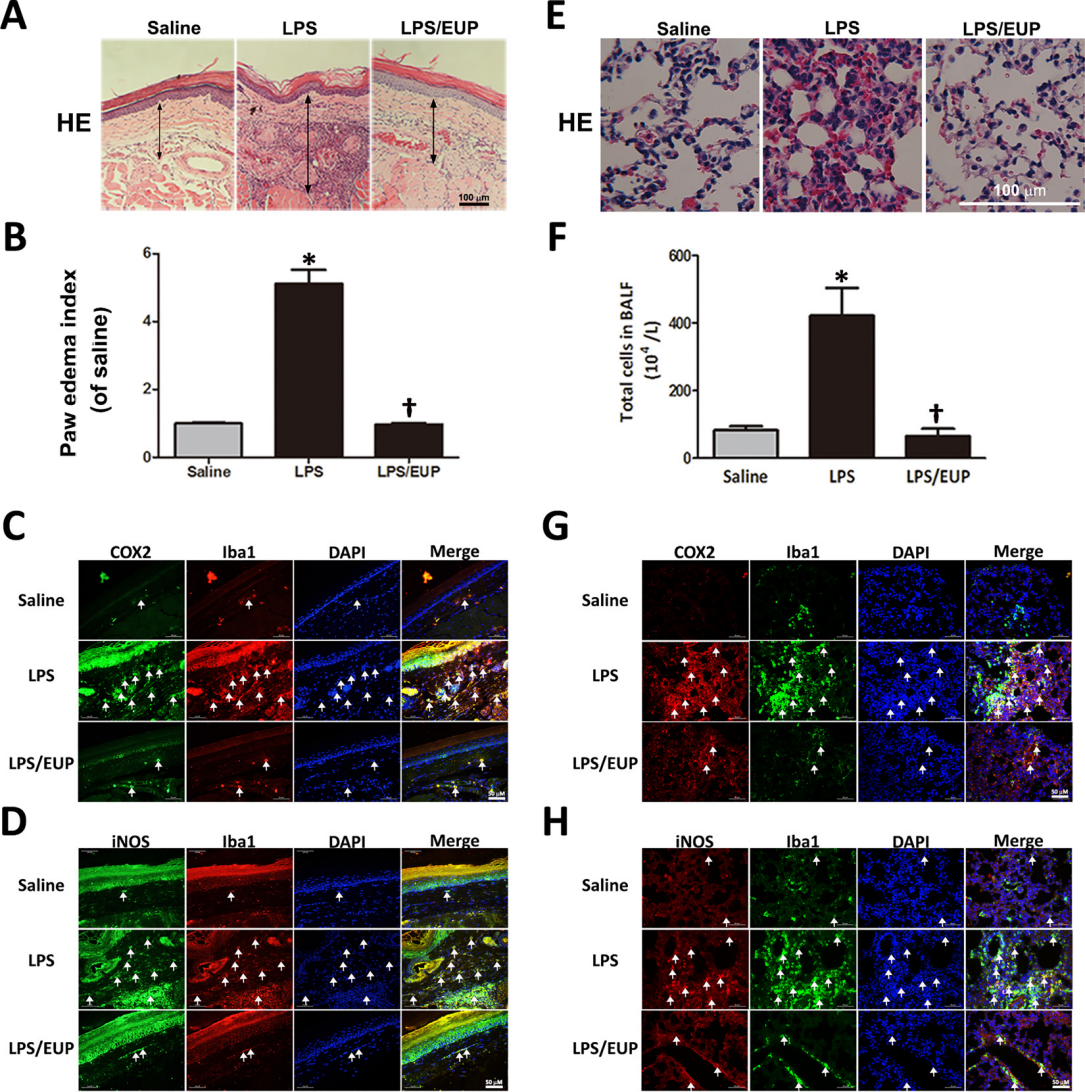
Eupafolin inhibited LPS-induced paw edema and inflammation in mice. (A) Representative cross-sections of the mouse paws (n = 4) were stained with hematoxylin and eosin. The degree of paw edema is shown between the two arrows. Bar = 100 μm. (B) Quantification of the degree of paw edema. The values are shown as the means ± SEM. *P<0.05 vs. the saline-treated mice. ^†^*P*<0.05 vs. the LPS-treated mice. (C-D) Immunohistochemical staining with the antibodies to COX-2, iNOS and Iba1 in paw sections. The positive reactions are indicated by arrowheads. Bar = 50 μm. Eupafolin inhibited the LPS-induced paw inflammation in mice. (E) Representative cross-sections of lungs were stained with hematoxylin and eosin. Bar = 50 μm. (F) Quantification of the degree of inflammatory cell infiltration into the lung. The values are shown as the means ± SEM. *P<0.05 vs. the saline-treated mice. ^†^*P*<0.05 vs. the LPS-treated mice. (G-H) Immunohistochemical staining with the antibodies to COX-2, iNOS and Iba1 in lung sections. The positive reactions are indicated by arrowheads. The sections were stained for COX-2 or iNOS (arrows) and Iba1 (macrophage marker, arrows) antibodies by double immunofluorescent staining. Nuclei were stained with DAPI (arrows). The stronger COX-2 or iNOS expression was present in the macrophage (arrows, the merged image). Bar = 50 μm.

To evaluate the effect of eupafolin on LPS-induced paw edema, we evaluated the paw-thickening changes that occurred in the paws of LPS-administered mice that were pretreated with or without eupafolin. Compared with those in the control group, the paw sections from the LPS-administered groups showed extensive morphological damage, including interstitial edema and infiltration of polymorphonuclear leukocytes (PMNs) into the paws (Fig 7A). LPS induced a remarkable increase in paw thickness resulting from edema, and this increase was effectively suppressed by pretreatment with eupafolin (*P*<0.05; Fig 7B) (from 5.11±0.42 to 0.97±0.04). Additionally, immunostaining of the paws showed that LPS increased the expression of iNOS and COX-2, and the stronger COX-2 and iNOS expression were closely associated with macrophage, which were identified by Iba1 (Fig 7C and 7D). In contrast, eupafolin pretreatment significantly suppressed the LPS-induced changes (Fig 7C and 7D).

### Effects of eupafolin on LPS-induced acute lung inflammation in mice

Animal pulmonary inflammation models induced by the experimental administration of LPS have been used extensively to test new anti-inflammatory drugs[23, 24]. The infiltration of inflammatory cells, especially neutrophils, into airspaces is a key event in the development of LPS-induced acute lung inflammation (ALI)[25]. To evaluate the effect of eupafolin on ALI, we examined the histopathological changes that occurred in the lungs of LPS-administered mice pretreated with or without eupafolin. In the control group, the lungs presented normal structure; no histopathological change was observed using light microscopy (Fig 7E). In contrast, the lung tissues from the LPS-administered groups without eupafolin pretreatment showed extensive morphological damage, including hemorrhage, interstitial edema, thickening of the alveolar walls, and infiltration of PMNs into the parenchyma and alveolar spaces of lung. Eupafolin pretreatment (EUP) for 30 min attenuated the LPS-induced histopathological damage. To determine whether eupafolin reduced the PMN infiltration, BALF was collected and analyzed by cell counting. Compared with the normal group (saline), the LPS-treated group (LPS) had substantially more PMNs in the BALF, and this increase was significantly reduced by eupafolin pretreatment (*P*<0.05; Fig 7F) (from 421.5±82.36 to 65.67±22.18). Analysis of COX-2 and iNOS revealed more COX-2- and iNOS-positive cells in the lung parenchyma of the LPS-administered group than in the lung parenchyma of the saline and LPS/EUP groups (Fig 7G and 7H). Similar effects were observed in the LPS-induced ALI experiments, the stronger COX-2 and iNOS expression were closely associated with macrophage (Iba1) (Fig 7G and 7H). These results indicated that eupafolin can improve the histopathological conditions in the lung caused by LPS-induced ALI.

## Discussion

The pathology of inflammation is initiated by complex processes triggered by microbial pathogens or their antigens. The prototypical endotoxin LPS can potently activate macrophages and induce various proinflammatory mediators; therefore, reducing activation signals in activated macrophages has been suggested as a therapeutic strategy for various inflammatory diseases.

In the present study, we found that eupafolin inhibited the LPS-induced production of NO and PGE_2_ by suppressing the transcription of inflammation-associated enzymes (iNOS and COX-2) and thus their protein expression. In addition to the inhibitory effect on these inflammation-related enzymes, the anti-inflammatory effects of eupafolin are consistent with the results of our investigations of its effects on several proinflammatory cytokines, namely, G-CSF, GM-CSF, IL-6, sICAM-1, MIP-2, IP-10, and TNF-ɑ, in LPS-induced macrophages. These cytokines have profound effects on the regulation of immune reactions, hematopoiesis, inflammation, and, in some cases, shock and death.

NF-κB and AP-1, important regulatory transcription factors, play pivotal roles in the regulation of the expression of iNOS, COX-2 and proinflammatory cytokines, such as TNF-α[20–22]. Upon activation, free NF-κB (a heterodimer of p50 and p65) and c-fos, a subunit of AP-1, pass into the nucleus, where they bind to the promoter regions of genes for inflammatory proteins, such as cytokines, enzymes, and adhesion molecules. Therefore, we examined the effect of eupafolin on the translocation of the p65 and c-fos subunits into the nucleus. Our results showed that eupafolin decreased the LPS-induced translocation of p65 and c-fos from the cytosol into the nucleus, and this effect was prevented by pretreatment with a JNK inhibitor. This finding is agreed with other data showing that JNK pathway is important signal in the activation of NF-κB and AP-1 in the processes of inflammatory reaction[26–28]. The present study showed that eupafolin inhibited the LPS-induced activation of NF-κB and AP-1 by suppressing the nuclear translocation of the p65 subunit and c-fos in RAW264.7 macrophages by western blot and by confocal microscope observation. And the inhibition mechanism may mainly through JNK inhibition for inactivation of NF-κB and AP-1, and thus inhibitory effects of eupafolin on the LPS -induced NO and PGE2 production.

The MAPK pathway plays important regulatory roles in the immune response[16–18]. MAPKs play an important role in the transcriptional regulation of the LPS-induced expression of iNOS and COX-2[29, 30]. Our results showed that the phosphorylation of JNK, ERK and p38 in response to LPS was reduced by eupafolin pretreatment. Additionally, the results demonstrated that the specific inhibitors of JNK and p38, but not the inhibitor targeting ERK, suppressed the expression of the iNOS and COX-2 genes. These results indicated the possibility that anti-inflammatory mechanism of eupafolin was mediated by the activation of the MAPK signaling pathway.

In addition to the *in vitro* results, we also observed that LPS-induced inflammation increased the thickness resulting the paw edema, the neutrophil accumulation in the lung and the expression of COX-2 and iNOS in the mouse paw and lung. Additionally, eupafolin effectively inhibited the expression of the inflammatory cytokines. Because it has been shown that infiltrating macrophages express COX-2 and iNOS proteins and secrete proinflammatory cytokines, our *in vivo* results suggest that eupafolin suppressed the LPS-induced inflammatory responses by inhibiting the proinflammatory cytokine production and the expression of COX-2 and iNOS in the infiltrating macrophages.

Macrophages play a critical role in the inflammatory process through the production of various cytokines[31]. Depending on the different forms of macrophage stimulation, these cells can be classified as M1 and M2 macrophages. M1, classically activated macrophages, express inflammation-related enzymes (iNOS and COX-2) and proinflammatory cytokines (TNF-α, IL-6, and MIP-1) when stimulated by LPS or IFN-γ. However, M2, alternatively activated macrophages, play an anti-inflammatory role in which IL-4, IL-13, or IL-10 is expressed[32]. Here, we demonstrated that eupafolin potently inhibited the LPS-induced expression of iNOS and COX-2 and the secretion of TNF-α, IL-6, MIP-1, RANTES, IP-10, G-CSF and GM-CSF. In addition to suppressing the expression of LPS-induced M1 mediators, eupafolin also increased the expression of CCL17 (TARC, a M2 marker)[33, 34] (Fig 2). These results suggest that eupafolin exerts its anti-inflammatory properties by inhibiting the expression of proinflammatory mediators, and this effect may be partly the result of polarization of the macrophage activation toward the M2 subtype rather than M1.

**Fig 8:**
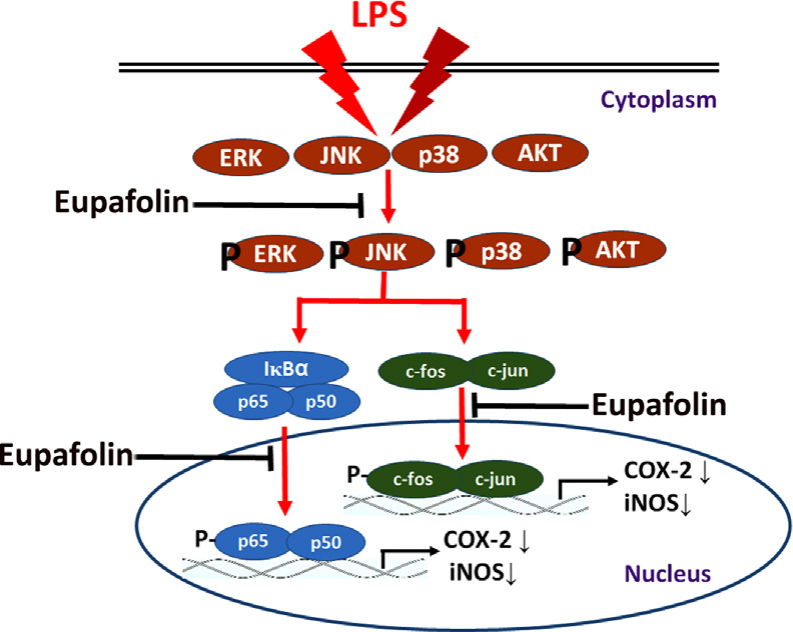
Graphical scheme of the anti-inflammatory mechanism of eupafolin in LPS-treated RAW264.7 macrophages.

The results of the present study clearly show that eupafolin derived from *Artemisia princeps* Pampanini exerts anti-inflammatory effects by inhibiting the production of NO and PGE_2_, as well as their upstream enzymes, iNOS and COX-2, at the protein level mainly through inhibition of JNK phosphorylation and NF-κB p65 and AP-1/c-fos activation in LPS-treated RAW264.7 cells (Fig 8); eupafolin also suppresses the LPS-induced ALI and paw inflammation in mice. These results indicate that eupafolin is a good candidate for use as an anti-inflammatory agent.
